# Diagnostic Accuracy of Ber-EP4 for Metastatic Adenocarcinoma in Serous Effusions: A Meta-Analysis

**DOI:** 10.1371/journal.pone.0107741

**Published:** 2014-09-17

**Authors:** Bo Wang, Diandian Li, Xuemei Ou, Qun Yi, Yulin Feng

**Affiliations:** Department of Respiratory Medicine, West China Hospital of Sichuan University, Chengdu, Sichuan, China; University of Utah School of Medicine, United States of America

## Abstract

Numerous studies have investigated the utility of Ber-EP4 in differentiating metastatic adenocarcinoma (MAC) from malignant epithelial mesothelioma (MM) and/or reactive mesothelial cells (RM) in serous effusions. However, the results remain controversial. The aim of this study is to determine the overall accuracy of Ber-EP4 in serous effusions for MAC through a meta-analysis of published studies. Publications addressing the accuracy of Ber-EP4 in the diagnosis of MAC were selected from the Pubmed, Embase and Cochrane Library. Data from selected studies were pooled to yield summary sensitivity, specificity, positive and negative likelihood ratio (LR), diagnostic odds ratio (DOR), and receiver operating characteristic (SROC) curve. Statistical analysis was performed by Meta-Disc 1.4 and STATA 12.0 softwares. 29 studies, based on 2646 patients, met the inclusion criteria and the summary estimating for Ber-EP4 in the diagnosis of MAC were: sensitivity 0.8 (95% *CI*: 0.78–0.82), specificity 0.94 (95% *CI*: 0.93–0.96), positive likelihood ratio (PLR) 12.72 (95% *CI*: 8.66–18.7), negative likelihood ratio (NLR) 0.18 (95% *CI*: 0.12–0.26) and diagnostic odds ratio 95.05 (95% *CI*: 57.26–157.77). The SROC curve indicated that the maximum joint sensitivity and specificity (Q-value) was 0.91; the area under the curve was 0.96. Our findings suggest that BER-EP4 may be a useful diagnostic adjunctive tool for confirming MAC in serous effusions.

## Introduction

Distinguishing metastatic adenocarcinoma (MAC) from malignant mesothelioma (MM) and/or reactive mesothelial cells (RM) is very important for staging and has significant treatment implications. However, it is difficult to differentiate malignant cells from reactive mesothelial cells, especially in cases involving malignant mesothelioma versus adenocarcinoma [1]–[4]. A biopsy provides a relatively high sensitivity and has been used as the gold standard diagnostic method [5], [6], however, these operations are invasive, operator dependent, and may complicate subsequent disease management by seeding tumor cells or be unfeasible because of poor condition of the patient. Tumor biomarkers are attractive adjuncts because of their noninvasive feature and relative inexpensiveness. So far, many tumor biomarkers directed against specific cell type antigens have been used in serous effusions to improve the accuracy of diagnosis, but the results are not always in agreement [7], [8]. It remains unclear which marker has a superior performance and application of a novel panel of diagnostic markers for early and accurate detection of MAC is mandatory to aid conventional tests.

Ber-EP4 is a monoclonal antibody that identifies 34-kD and 39-kD cell surface glycoproteins present on the membrane of human epithelial cells but not on reactive or malignant mesothelial cells [9]. An increasing number of studies have shown the ability of this antibody to be a marker in the differential diagnosis of MAC from MM/RM [1], [10]–[37]. Systematic analysis of these data may be valuable to finally confirm the application potential of Ber-EP4 as a marker for MAC. So we performed this meta-analysis to explore the potential value of Ber-EP4 in the diagnosis of MAC from MM/RM, which, to the best of our knowledge, has not been previously performed.

## Materials and Methods

### Search strategy and study selection

A search of the literature was conducted using the electronic databases Pubmed, Embase, Cochrane Library, Web of Science, and The Chinese Journals Full-text Database (CNKI) (updated to December 31, 2013). The search terms used were: “Ber-EP4,” “body fluids,” “effusions,” “sensitivity and specificity,” and “accuracy.” Only full-text papers published in English and Chinese were included. The reference lists of all articles reviewed were also searched for eligible studies. The following criteria were used in the selection of literature for meta-analysis: (1) studies evaluated Ber-EP4 in the differential diagnosis of MAC and MM/RM in serous effusions, (2) each study contains more than ten fluid specimens, and (3) studies must provide sufficient data to calculate both sensitivity and specificity. Publications with evidence of a possible overlap of patients with other studies were discussed by BW and DDL and only the best quality study was used. Two reviewers (BW and DDL) independently judged study eligibility while screening the citations. Disagreements were resolved by consensus.

### Data extraction and quality assessment

Two authors (BW and DDL) independently extracted the data and reached a consensus on all items. Any discrepancies were resolved by discussion with a third author (YLF) to reach a final consensus. The following data were collected from each study: the first author’s name, publication year, country, test methods, cutoff value, sensitivity, specificity. The methodological quality of each study was assessed using guidelines published by the STARD (standards for reporting diagnostic accuracy, maximum score 25) initiative [38] (ie, guidelines that aim to improve the quality of reporting in diagnostic studies) and the QUADAS-2 (quality assessment for studies of diagnostic accuracy, an evidence-based quality assessment tool for use in systematic reviews of diagnostic accuracy studies) tool. The QUADAS-2 tool consists of 4 key domains that discuss patient selection, index test, reference standard and flow of patients through the study and timing of the index tests and reference standard (flow and timing) [39].

### Statistical analyses

The standard methods recommended for the diagnostic accuracy of meta-analyses were used [40]. The following indexes of test accuracy were computed for each study: sensitivity, specificity, positive likelihood ratio (PLR), negative likelihood ratio (NLR), and diagnostic odds ratio (DOR). The diagnostic threshold identified for each study was used to plot a summary receiver operating characteristic (SROC) curve [41]. To detect cut-off threshold effects, the relationship between sensitivity and specificity was evaluated by the Spearman correlation coefficient. The inter-study heterogeneity was calculated by the chi-square-based Q test and the inconsistency index I^2^. When a significant Q test (p<0.05 or I^2^>50%) indicated heterogeneity among studies, the random-effect model (DerSimonian–Laird method) was conducted for the meta-analysis to calculate the pooled sensitivity, specificity, and other related indexes of the studies; otherwise, the fixed-effect model (Mantel–Haenszel method) was chosen. Meta-regression was performed to investigate the source of heterogeneity within the included studies (inverse variance weighted) [42]. Since publication bias is of concern for meta-analyses of diagnostic studies, we tested for the potential presence of this bias using Deeks’ funnel plots [43]. Analyses were performed using the following statistical software programs: STATA, version 12.0 (Stata Corporation, College Station, TX, USA) and Meta-Disc 1.4 for Windows (XI Cochrane Colloquium, Barcelona, Spain) [44], [45]. In every test, a two-sided p-value of <0.05 was considered statistically significant.

## Results

### Quality of reporting and study characteristics

**Figure 1:**
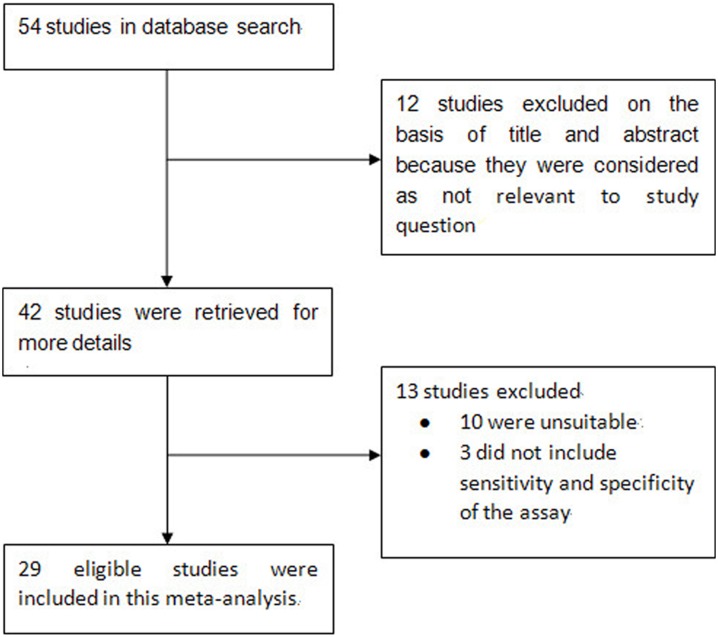
Flow chart of selection process for eligible articles.

The article selection process used in this study is summarized in Fig. 1. A total of 29 studies published between 1993 and 2013 met the inclusion criteria and were included in the present meta-analysis. The main clinical characteristics of the included studies are presented in Table 1. Overall, the 29 selected studies, which originated from 14 countries, included 2646 individuals and the sample size varied from 17 to 232 individuals with an average size of 90 individuals. In all studies included in the meta-analysis, the cytological diagnoses of all cases were proved by histopathology or clinical data. 19 of all studies received the same reference standard, indicating that there was partial potential verification bias. 21 of all studies, samples were collected from consecutive or random selected patients. 6 of all studies make inappropriate exclusions (for example, not including “difficult-to-diagnose” patients). Only 1 study did not report blinded interpretation of Ber-EP4 assay independent of the reference standard. Most studies had an adequate description of the used cut-off value of the marker. Details of the staining methods of the studies included in the meta-analysis was presented in Table S1. In all, 29 studies included in our meta-analysis had higher STARD scores (≥13, data not shown), which showed high quality. As shown in Table 2, validity of included trials was assessed using the QUADAS-2 tool. Based on the methods reported in each trial, each of the 14 components according to QUADAS-2 criteria was graded ‘‘yes’’, ‘‘unclear’’ or ‘‘no’’, which meant ‘‘low risk of bias’’, ‘‘uncertain of bias’’ and ‘‘high risk of bias’’, respectively [39].

**Table 1:** Summary of the studies included in the meta-analysis.

First author-year	Country	Method	Cutoff	Sample Size	TP	FP	FN	TN
Diaz-Arias AA - 1993 [10]	Columbia	Cell blocks	≥10% cellsstained	232	85	3	18	126
Illingworth AL - 1994 [11]	UK	Smears	Membranousand/orcytoplasmicstaining	42	23	0	3	16
Shield PW - 1994 [12]	Australia	Cell blocks	Membranousand/orcytoplasmic staining	153	33	0	69	51
Matter Walstra KW - 1996 [13]	Switzerland	Smears	Membranousand/orcytoplasmic staining	66	28	0	10	28
Bailey ME - 1996 [14]	America	Cell blocks	Membranous staining	32	11	0	0	21
Jensen ML - 1996 [15]	Denmark	Cell blocks	Membranous and/or cytoplasmic staining	94	24	2	10	58
Delahaye M - 1997 [16]	Netherlands	Smears	Membranousand/orcytoplasmicstaining	154	69	1	19	65
Nagel H - 1998 [17]	Germany	Smears	Membranousand/orcytoplasmicstaining	107	34	12	8	53
Motherby H - 1999 [18]	Germany	Cell blocks	≥5% cellsstained	64	35	0	10	19
Bjorn Risberg - 2000 [19]	Norway	Cell blocks	Membranousand/orcytoplasmicstaining	29	16	0	1	12
Dejmek A - 2000 [20]	Sweden	Smears	Membranousand/orcytoplasmicstaining	113	51	8	2	52
Davidson B - 2001 [21]	Norway	Cell blocks	Membranousand/orcytoplasmicstaining	166	94	8	4	60
Xiangju Li - 2005 [22]	China	Cell blocks	≥5% cellsstained	150	81	0	36	33
Alaa Afify - 2005 [23]	America	Cell blocks	Membranousstaining	64	33	0	6	25
Politi E - 2005 [24]	Greece	Smears	≥10% cellsstained	134	62	0	18	54
Wanxin W - 2005 [25]	China	Cell blocks	Membranousand/orcytoplasmicstaining	80	47	0	11	22
Dejmek A - 2005 [26]	Sweden	Smears	≥30% cellsstained	104	77	3	8	16
Aerts JG - 2006 [27]	Netherlands	Smears	Membranousand/orcytoplasmicstaining	39	12	1	0	26
Fang F - 2006 [28]	China	Cell blocks	≥10% cellsstained	86	38	3	5	40
Ueda J - 2006 [1]	Japan	Cell blocks	Membranousand/orcytoplasmicstaining	17	5	0	8	4
Johanna M - 2007 [29]	Netherlands	Cell blocks	Membranousand/orcytoplasmicstaining	34	11	0	1	22
Palaoro LA - 2007 [30]	Argentina	Smears	Membranousand/orcytoplasmicstaining	45	16	1	9	19
Saleh HA - 2009 [31]	America	Cell blocks	≥5% cellsstained	84	34	2	7	41
Bing Liu - 2010 [32]	China	Smears	≥10% cellsstained	180	135	0	15	30
McKnight R - 2010 [33]	America	Cell blocks	≥5% cellsstained	82	29	7	12	34
Su XY - 2011 [34]	China	Cell blocks	Membranousand/orcytoplasmicstaining	93	42	5	13	33
Mingzhi C- 2011 [35]	China	Cell blocks	Membranousand/orcytoplasmicstaining	30	23	0	3	4
Arora R - 2011 [36]	India	Cell blocks	Membranousand/orcytoplasmicstaining	100	49	7	1	43
Yingcheng T - 2012 [37]	China	Cell blocks	Cytoplasmicstaining	72	28	0	0	44

TP, true positive; FP, false positive; FN, false negative; TN, true negative.

**Table 2:** Summary of the methodological quality assessment of the included studies according to QUADAS-2 criteria.

Studies	Risk of Bias				Applicability concerns		
	Patient Selection	Index Test	Reference Standard	Flowand Timing	Patient Selection	Index Test	Reference Standard
Diaz-Arias AA - 1993	LR	LR	UR	UR	LC	LC	UC
Illingworth AL - 1994	LR	LR	LR	UR	LC	UC	LC
Shield PW - 1994	UR	LR	LR	LR	LC	LC	LC
MatterWalstra KW- 1996	LR	LR	UR	HR	LC	UC	LC
Bailey ME - 1996	UR	UR	UR	LR	LC	LC	LC
Jensen ML - 1996	LR	LR	LR	LR	LC	LC	LC
Delahaye M - 1997	HR	LR	LR	LR	LC	UC	LC
Nagel H - 1998	UR	LR	UR	UR	LC	UC	LC
Motherby H - 1999	LR	LR	UR	UR	LC	LC	UC
BjornRisberg - 2000	LR	LR	LR	UR	UC	LC	LC
Dejmek A - 2000	LR	LR	LR	LR	LC	UC	LC
Davidson B - 2001	LR	LR	UR	UR	LC	LC	LC
Xiangju Li - 2005	LR	LR	UR	LR	UC	LC	LC
Alaa Afify - 2005	HR	LR	LR	LR	LC	LC	LC
Politi E - 2005	LR	LR	LR	LR	LC	UC	LC
Wanxin W - 2005	HR	UR	LR	UR	LC	LC	LC
Dejmek A - 2005	LR	LR	LR	UR	LC	UC	LC
Aerts JG - 2006	HR	LR	LR	LR	UC	UC	LC
Fang F - 2006	UR	LR	LR	LR	LC	LC	LC
Ueda J - 2006	LR	LR	LR	UR	LC	LC	LC
Johanna M - 2007	HR	LR	LR	LR	LC	LC	LC
Palaoro LA - 2007	UR	LR	LR	UR	LC	UC	LC
Saleh HA - 2009	HR	LR	LR	LR	LC	LC	LC
Bing Liu - 2010	LR	LR	UR	LR	LC	UC	LC
McKnight R - 2010	UR	LR	UR	UR	LC	LC	UC
Su XY - 2011	UR	LR	LR	UR	LC	LC	LC
Mingzhi C- 2011	LR	LR	UR	UR	UC	LC	UC
Arora R - 2011	LR	LR	LR	LR	LC	LC	LC
YingchengT - 2012	LR	LR	UR	UR	UC	LC	LC

LR: low risk; HR: high risk; UR: unclear risk; LC: low concern; HC: high concern; UC: unclear concern.

### Diagnostic accuracy

The between-study heterogeneity was assessed by *I^2^* index to choose the appropriate calculation model. The *I^2^* of sensitivity, specificity, positive likelihood ratio (PLR), negative likelihood ratio (NLR) and DOR were 88.1% (*p*<0.0001), 69.2% (*p*<0.0001), 52.2% (*p* = 0.0006), 92.5% (*p*<0.0001), and 44.2% (*p* = 0.0061), respectively. Therefore, the random effects model was used for calculating pooled sensitivity, specificity, PLR, NLR and DOR in present meta-analysis.

**Figure 2:**
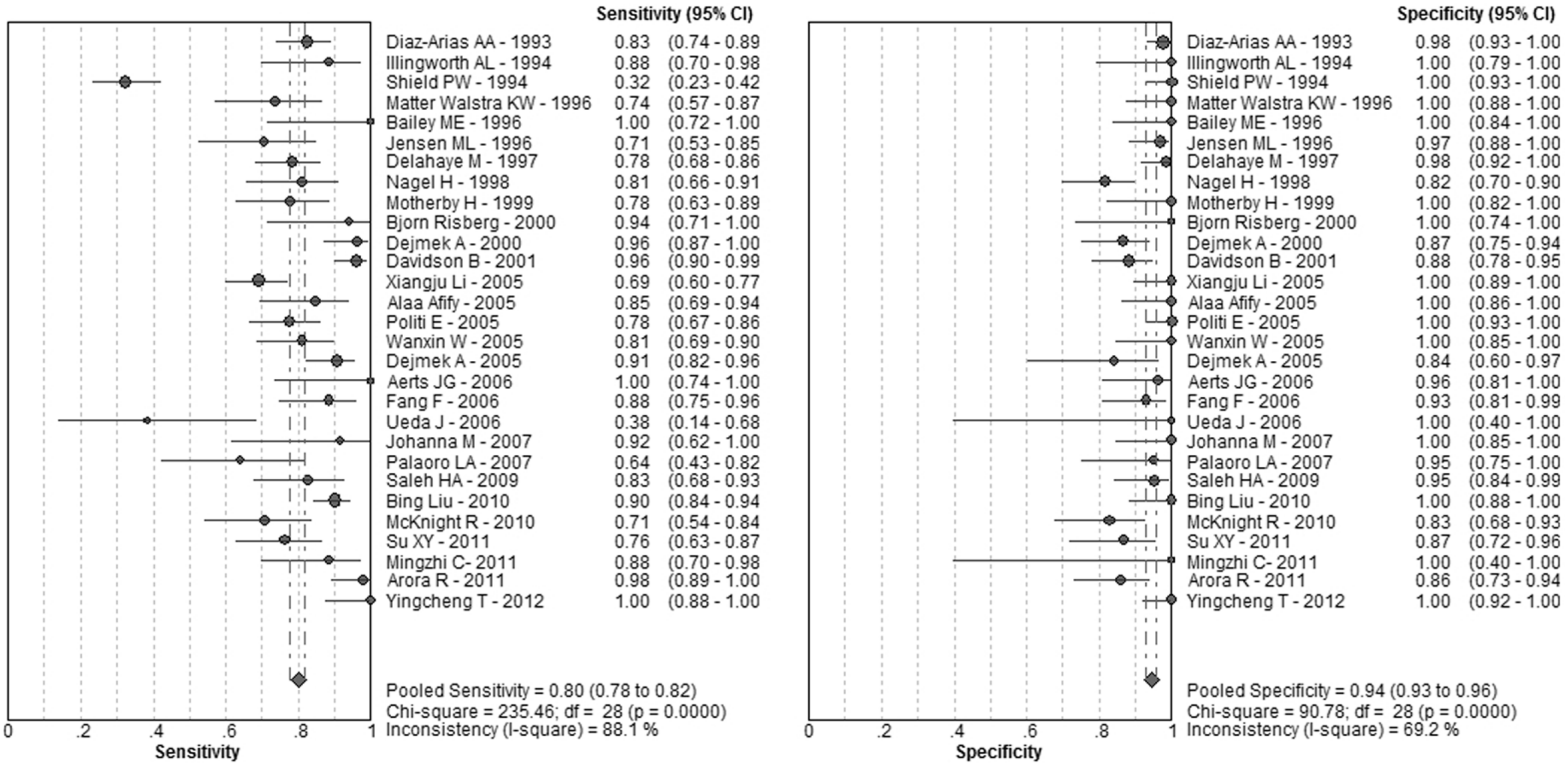
Forest plots of the sensitivity and speci­ficity for Ber-EP4 in the diagnosis of metastatic adenocarcinoma for all studies. The point estimates of sensitivity and specificity for each study are shown as solid circles and the size of each solid circle indicates the sample size of each study. Error bars are 95% confidence intervals.

**Figure 3:**
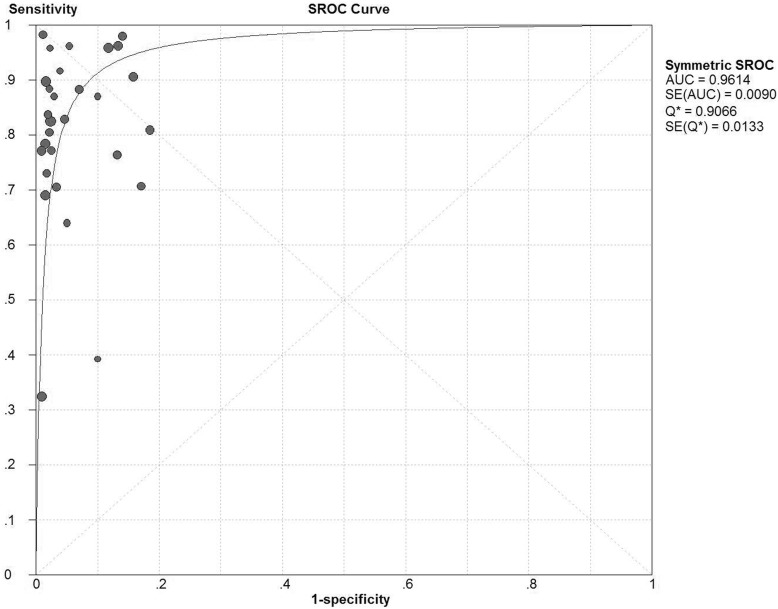
Summary receiver operating characteristic curve for Ber-EP4 in the diagnosis of metastatic adenocarcinoma for all studies. Solid circles represent each study included in the meta-analysis. The size of each solid circle indicates the size of each study. The regression SROC curve summarizes the overall diagnostic accuracy.

Fig. 2 shows the forest plots of the sensitivity and speci­ficity of these 29 studies concerning BerEP4 in the diagnosis of MAC. The pooled sensitivity and specificity were 0.8 (95% *CI*: 0.78–0.82) and 0.94 (95% *CI*: 0.93–0.96), respectively. The overall PLR and NLR were 12.72 (95% *CI*: 8.66–18.7) and 0.18 (95% *CI*: 0.12–0.26), respectively. The pooled diagnostic odds ratio (DOR) was 95.05 (95% CI: 57.26–157.77). The SROC curve for BerEP4 is shown in Fig. 3, which indicates sensitivity versus 1-specificity of individual studies. As a global measure of test efficacy we used Q-value, the intersection point of the SROC curve with a diagonal line from the left upper corner to the right lower corner of the ROC space which corresponds to the highest common value of sensitivity and specificity for the test, for the overall measure of the discriminatory power of the test. Our data showed that the SROC curve for BerEP4 is positioned near the desirable upper left corner and the Q-value was 0.91; while the area under the curve (AUC) was 0.96, indicating that the level of overall accuracy was high. To explore the possible reasons for the heterogeneity, a meta-regression analysis based on test method (cell blocks or smears), sample size (≥100 or <100), lack of blind and other methodological quality according to QUADAS-2 tool and STARD guideline (data not shown). Statistical significance could be observed between studies with and without enrolling consecutive/random sample of patients (*p* = 0.0002, data not shown). None of the other covariates included in the meta-regression was found to be the significant source of heterogeneity (all *p*>0.05, data not shown).

### Publication bias evaluation

**Figure 4:**
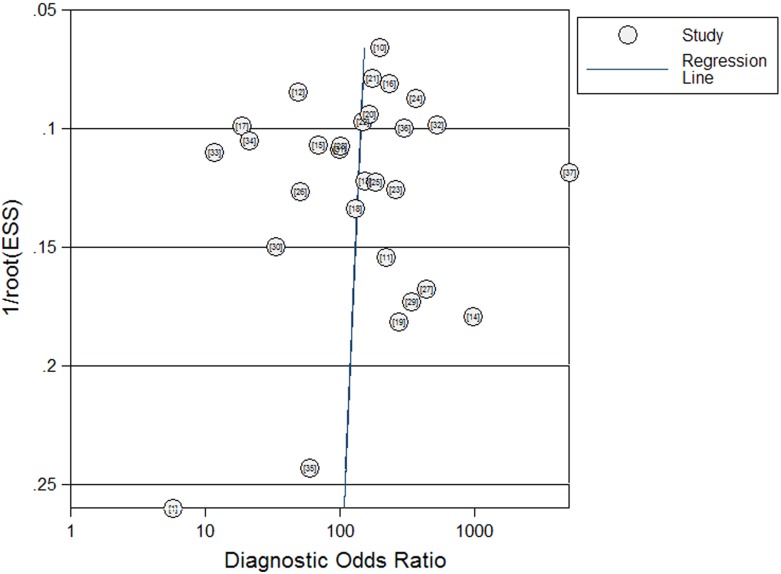
Funnel graph for the assessment of potential publication bias of the 29 included studies. The funnel graph plots the log of the diagnostic odds ratio (DOR) against the standard error of the log of the DOR (an indicator of sample size). Solid circles represent each study in the meta-analysis. The line indicates the regression line.

Publication bias was explored through Deeks’ funnel plots. The shape of the funnel plot of the pooled DOR of BerEP4 for the diagnosis of malignant effusions did not reveal any evidence of obvious asymmetry (Fig. 4). The Deeks’ test also showed a statistically non-significant value (*p* = 0.81), indicating that there was no potential publication bias.

## Discussion

Effusion in body cavities is a common complication which may result from a variety of clinical settings including infections, cardiac failure, and malignancies such as lung, breast, gastrointestinal, and female genital adenocarcinoma as well as malignant mesothelioma [46], [47]. Distinguishing malignant epithelial cells from mesothelial cells is critical in the differential diagnosis of body cavity effusions. However, adenocarcinoma metastatic to serous membranes is often associated with prominent mesothelial hyperplasia and often results in diagnostic confusion. This phenomenon is a major problem in routine cytology, and a reliable method is needed. Immunohistochemistry can greatly aid in resolving such diagnostic dilemmas. Unfortunately, currently available markers have varying sensitivities and specificities for epithelial or mesothelial cells. Ber-EP4 is a monoclonal antibody that identifies 34-kD and 39-kD cell surface glycoproteins present on the membrane of human epithelial cells but not on reactive or malignant mesothelial cells. In recent years, an increasing number of studies have attempted to evaluate the diagnostic accuracy of Ber-EP4 for MAC but the results remain controversial because of several factors, including the differences in study designs, sample size, statistical methods, etc. [48]. In this regard, we performed this current meta-analysis to comprehensively assess the diagnostic accuracy of Ber-EP4 for MAC in serous effusions.

The SROC curve presents a global summary of test performance, and shows the trade-off between sensitivity and specificity. The present meta-analysis has shown that the mean sensitivity of the Ber-EP4 was 0.8 while the mean specificity was 0.94, and that the maximum joint sensitivity and specificity (Q value) was 0.91 while the AUC was 0.96, indicating a good overall accuracy in the diagnosis of MAC, although not perfect. The DOR, the ratio of the odds of positivity in disease relative to the odds of positivity in the non-diseased, is a single indicator of diagnostic test performance [49] that combines the data from sensitivity and specificity into a single number. The value of a DOR ranges from 0 to infinity, with higher values indicating better discriminatory test performance (higher accuracy). A DOR of 1.0 indicates that a test cannot discriminate between patients with the disorder and those without it. In this meta-analysis, the pooled DOR was 95.05, also suggesting a high level of overall accuracy. However, the SROC curve and the DOR are not easy to interpret and use in clinical practice, while the likelihood ratio (PLR and NLR) is more clinically meaningful for our measures of diagnostic accuracy. A PLR value of 12.72 suggests that patients with MAC have about 13-fold higher chance of being Ber-EP4-positive compared to those with MM/RM, and this was high enough for the clinical practice. On the other hand, the NLR was 0.18, which means that the probability of having MAC in Ber-EP4-negative patients is 18% in theory, while, for instance, cancer cells may be absent or scanty on the cell blocks or smears used for immunostaining, which may have inflated the false negative rate.

The *I^2^* test for the pooled sensitivity, specificity, PLR, NLR and DOR showed that the heterogeneity between the studies was obvious. So we undertook a meta-regression analysis to find the possible reasons for heterogeneity. Some papers have reported that the cell block sections may be the most suitable form of sample preparation when performing immunostaining on effusions due to ease of morphologic interpretation, standardized like-like comparison with surgical pathology material, least amount of background stain, and expected immunostaining patterns [32]–[35]. So we first considered that the test method (cell blocks or smears) might contribute to the heterogeneity. However, meta-regression analysis indicates that the above variable was not the source of heterogeneity (*p* = 0.9046, data not shown). The other primary cause of heterogeneity in test accuracy studies is threshold effect, which arises when differences in sensitivities and specificities occur due to different cut-offs or thresholds used in different studies to define a positive or negative test result. We used the Spearman correlation coefficient to analyze the threshold effect. No heterogeneity could be observed from threshold effects (*p*>0.05, data not shown). Then we chose to investigate whether the QUADAS results, the STARD scores, lack of blinding, and the sample size were responsible for the heterogeneity noted. Statistical significance was observed between studies with and without enrolling consecutive/random sample of patients (*p* = 0.0002), indicating that patient selection bias may affect the diagnostic accuracy. The study participants must be representative of the study entrants in order for the study participants. Therefore, a study ideally should enroll a consecutive or random sample of eligible patients with suspected disease to prevent the potential patient selection bias [39], [50].

In the present meta-analysis, the results indicate that Ber-EP4 may, to a certain extent, be valuable in the differential diagnosis of MAC in serous effusions. However, no single marker alone can establish the diagnosis in all cases of body cavity fluid, and combinations of Ber-EP4 with other epithelial or mesothelial stains are recommended to increase diagnostic accuracy [24]. It has been reported that MOC-31 was100% sensitive and 100% specific in differentiating MAC from MM and RM, and the staining combination of positive for MOC-31 and negative for D2–40 or calretinin was 100% specific and 99% sensitive for MAC [24]. Furthermore, the combined use of Ber-EP4, MOC-31, CA19-9, and CEA antibodies might be a suitable panel for the discrimination between adenocarcinoma cells and reactive mesothelial cells [1]. However, due to the varying degrees of diagnostic accuracy of identical markers reported in different studies, it remains unclear which marker has a superior performance. Therefore, more immunomarkers should be comprehensively evaluated for their diagnostic accuracy and high-quality diagnostic tests are needed to find the optimum panel of antibodies for the diagnosis of MAC in serous effusions.

Our study had some limitations. First, only published studies were included in this meta-analysis, the exclusion of unpublished data, ongoing studies, conference abstracts and letters to editors may have led to publication bias. Second, verification bias can occur since some adenocarcinoma was diagnosed in some patients based just on the clinical course, but not diagnosed by histological examination. This issue regarding accuracy of diagnosis can cause nonrandom misclassification, leading to biased results. Furthermore, 6 studies that make inappropriate exclusions (for example, not including “difficult-to-diagnose” patients) may result in overestimation of diagnostic accuracy, even though no significance could be detected in our meta-regression analysis. Third, different cutoff values were used in the included studies, which made it difficult to determine the optimized cutoff value. Fourth, because of lacking of required data reported in the original publications, we could not analyse the effect of factors such as laboratory infrastructure, expertise with tumour marker assay technology, patient spectrum and setting on the accuracy of the Ber-EP4 measurements.

Despite these limitations, our study is the first comprehensive meta-analysis to date to have assessed the diagnostic accuracy of Ber-EP4 for MAC in serous effusions. The results demonstrated that Ber-EP4 may be a useful adjunct to conventional diagnostic tools for accurately differentiating MAC and MM/RM, but should be interpreted in parallel with the gold standard of morphology and clinical findings. Further blinded larger-scale prospective cohort studies are needed and they should focus on the application of a novel panel of diagnostic markers for early and accurate detection of MAC.

## Supporting Information

Table S1 **Summary of the studies included in the meta-analysis.**
(DOCX)

Checklist S1 **PRISMA checklist.**
(DOC)

## References

[1] Ueda J, Iwata T, Ono M, Takahashi M (2006) Comparison of three cytologic preparation methods and immunocytochemistries to distinguish adenocarcinoma cells from reactive mesothelial cells in serous effusion. Diagn Cytopathol 34: 6–10. 16355377 10.1002/dc.20391

[2] Butnor KJ (2006) My approach to the diagnosis of mesothelial lesions. J Clin Pathol 59: 564–574. 16731600 10.1136/jcp.2005.029652 PMC1860395

[3] Bedrossian CW (1998) Diagnostic problems in serous effusions. Diagn Cytopathol 19: 131–137. 9702493 10.1002/(sici)1097-0339(199808)19:2<131::aid-dc14>3.0.co;2-g

[4] Lyons-Boudreaux V, Mody DR, Zhai J, Coffey D (2008) Cytologic malignancy versus benignancy: how useful are the “newer” markers in body fluid cytology? Arch Pathol Lab Med 132: 23–28. 18181669 10.5858/2008-132-23-CMVBHU

[5] Kastelik JA (2013) Management of malignant pleural effusion. Lung 191: 165–175. 23315213 10.1007/s00408-012-9445-1

[6] Fassina A, Fedeli U, Corradin M, Da Frè M, Fabbris L (2008) Accuracy and reproducibility of pleural effusion cytology. Leg Med 10: 20–25. 10.1016/j.legalmed.2007.06.001 17702624

[7] Afify AM, Al-Khafaji BM, Paulino AF, Davila RM (2002) Diagnostic use of muscle markers in the cytologic evaluation of serous fluids. Appl Immunohistochem Mol Morphol 10: 178–182. 12051638 10.1097/00129039-200206000-00014

[8] Queiroz C, Barral-Netto M, Bacchi CE (2001) Characterizing subpopulations of neoplastic cells in serous effusions. The role of immunocytochemistry. Acta Cytol 45: 18–22. 11213499 10.1159/000327182

[9] Lozano MD, Panizo A, Toledo GR, Sola JJ, Pardo-Mindán J (2001) Immunocytochemistry in the differential diagnosis of serous effusions: A comparative evaluation of eight monoclonal antibodies in papanicolaou stained smears. Cancer 93: 68–72. 11241268

[10] Diaz-Arias AA, Loy TS, Bickel JT, Chapman RK (1993) Utility of BER-EP4 in the diagnosis of adenocarcinoma in effusions: an immunocytochemical study of 232 cases. Diagn Cytopathol 9: 516–521. 8287759 10.1002/dc.2840090509

[11] Illingworth AL, Young JA, Johnson GD (1994) Immunofluorescent staining of metastatic carcinoma cells in serious fluid with carcinoembryonic antibody, epithelial membrane antibody, AUA-1 and Ber-EP4. Cytopathology 5: 270–281. 7819512 10.1111/j.1365-2303.1994.tb00431.x

[12] Shield PW, Callan JJ, Devine PL (1994) Markers for metastatic adenocarcinoma in serous effusion specimens. Diagn Cytopathol 11: 237–245. 7532566 10.1002/dc.2840110309

[13] Matter-Walstra KW, Kraft R (1996) Atypical cells in effusions: diagnostic value of cell image analysis combined with immunocytochemistry. Diagn Cytopathol 15: 263–269. 8982578 10.1002/(SICI)1097-0339(199611)15:4<263::AID-DC3>3.0.CO;2-F

[14] Bailey ME, Brown RW, Mody DR, Cagle P, Ramzy I (1996) Ber-EP4 for differentiating adenocarcinoma from reactive and neoplastic mesothelial cells in serous effusions. Comparison with carcinoembryonic antigen, B72.3 and Leu-M1. Acta Cytol 40: 1212–1216. 8960030 10.1159/000333982

[15] Jensen ML, Johansen P (1996) Immunocytochemical staining of smears and corresponding cell blocks from serous effusions: a follow-up and comparative investigation. Diagn Cytopathol 15: 33–36. 8807249 10.1002/(SICI)1097-0339(199607)15:1<33::AID-DC7>3.0.CO;2-R

[16] Delahaye M, van der Ham F, van der Kwast TH (1997) Complementary value of five carcinoma markers for the diagnosis of malignant mesothelioma, adenocarcinoma metastasis, and reactive mesothelium in serous effusions. Diagn Cytopathol 17: 115–120. 9258618 10.1002/(sici)1097-0339(199708)17:2<115::aid-dc6>3.0.co;2-f

[17] Nagel H, Hemmerlein B, Ruschenburg I, Hüppe K, Droese M (1998) The value of anti-calretinin antibody in the differential diagnosis of normal and reactive mesothelia versus metastatic tumors in effusion cytology. Pathol Res Pract 194: 759–764. 9842634 10.1016/S0344-0338(98)80065-4

[18] Motherby H, Friedrichs N, Kube M, Nadjari B, Knops K, et al (1999) Immunocytochemistry and DNA-image cytometry in diagnostic effusion cytology. II. Diagnostic accuracy in equivocal smears. Anal Cell Pathol 19: 59–66. 10746435 10.1155/1999/934146 PMC4618576

[19] Risberg B, Davidson B, Dong HP, Nesland JM, Berner A (2000) Flow cytometric immunophenotyping of serous effusions and peritoneal washings: comparison with immunocytochemistry and morphological findings. J Clin Pathol 53: 513–517. 10961174 10.1136/jcp.53.7.513 PMC1731234

[20] Dejmek A, Hjerpe A (2000) Reactivity of six antibodies in effusions of mesothelioma, adenocarcinoma and mesotheliosis: stepwise logistic regression analysis. Cytopathology 11: 8–17. 10714371 10.1046/j.1365-2303.2000.00211.x

[21] Davidson B, Nielsen S, Christensen J, Asschenfeldt P, Berner A, et al (2001) The role of desmin and N-cadherin in effusion cytology: a comparative study using established markers of mesothelial and epithelial cells. Am J Surg Pathol 25: 1405–1412. 11684957 10.1097/00000478-200111000-00008

[22] Li XJ, Pan QJ, Shen GH, Liu XY, Sun YT (2005) Differential diagnostic value of B72.3, Ber-EP4 and calretinin in serous effusions. Zhonghua Zhong Liu Za Zhi 27: 438–441. 16188134

[23] Afify A, Zhou H, Howell L, Paulino AF (2005) Diagnostic utility of GLUT-1 expression in the cytologic evaluation of serous fluids. Acta Cytol 49: 621–626. 16450901 10.1159/000326249

[24] Politi E, Kandaraki C, Apostolopoulou C, Kyritsi T, Koutselini H (2005) Immunocytochemical panel for distinguishing between carcinoma and reactive mesothelial cells in body cavity fluids. Diagn Cytopathol 32: 151–155. 15690338 10.1002/dc.20203

[25] Wu WX, Wen XW, Lu N, Zhang YP, Qian JY, et al (2005) Value of E-cadherin in labeling carcinoma cell in body cavity effusions. Journal of Practical Oncology 5: 79.

[26] Dejmek A, Hjerpe A (2005) The combination of CEA, EMA, and BerEp4 and hyaluronan analysis specifically identifies 79% of all histologically verified mesotheliomas causing an effusion. Diagn Cytopathol 32: 160–166. 15690331 10.1002/dc.20202

[27] Aerts JG, Delahaye M, van der Kwast TH, Davidson B, Hoogsteden HC, et al (2006) The high post-test probability of a cytological examination renders further investigations to establish a diagnosis of epithelial malignant pleural mesothelioma redundant. Diagn Cytopathol 34: 523–527. 16850492 10.1002/dc.20486

[28] Fang F, Zhang W, Yang L, Su XL, He Q, et al (2006) Cytopathological differential diagnosis between reactive mesothelial cells and metastatic adenocarcinoma in serous effusion. J Diag Pathol 13: 3.

[29] Grefte JM, de Wilde PC, Salet-van de Pol MR, Tomassen M, Raaymakers-van Geloof WL, et al (2008) Improved identification of malignant cells in serous effusions using a small, robust panel of antibodies on paraffin-embedded cell suspensions. Acta Cytol 52: 35–44. 18323273 10.1159/000325432

[30] Palaoro LA, Blanco AM, Gamboni M, Rocher AE, Rotenberg RG (2007) Usefulness of ploidy, AgNOR and immunocytochemistry for differentiating benign and malignant cells in serous effusions. Cytopathology 18: 33–39. 17250601 10.1111/j.1365-2303.2007.00404.x

[31] Saleh HA, El-Fakharany M, Makki H, Kadhim A, Masood S (2009) Differentiating reactive mesothelial cells from metastatic adenocarcinoma in serous effusions: the utility of immunocytochemical panel in the differential diagnosis. Diagn Cytopathol 37: 324–332. 19191294 10.1002/dc.21006

[32] Liu B, Li YD, Zheng JY, Wang MN (2010) Differential diagnostic value of BerEP4, E-cadher in and Calretinin for benign and malignant pleuroperitoneal fluid. J Shanxi Med Univ 41: 10.

[33] McKnight R, Cohen C, Siddiqui MT (2010) Utility of paired box gene 8 (PAX8) expression in fluid and fine-needle aspiration cytology: an immunohistochemical study of metastatic ovarian serous carcinoma. Cancer Cytopathol 118: 298–302. 20572292 10.1002/cncy.20089

[34] Su XY, Li GD, Liu WP, Xie B, Jiang YH (2011) Cytological differential diagnosis among adenocarcinoma, epithelial mesothelioma, and reactive mesothelial cells in serous effusions by immunocytochemistry. Diagn Cytopathol 39: 900–908. 20836004 10.1002/dc.21489

[35] Chen MZ, Wang GZ (2011) ThinPrep Cytological Test combined with Immunocytochemistry in Diagnosis of Complicate Pleural Effusion Application. Chinese Journal of General Practice 9: 10.

[36] Arora R, Agarwal S, Mathur SR, Verma K, Iyer VK, et al (2011) Utility of a limited panel of calretinin and Ber-EP4 immunocytochemistry on cytospin preparation of serous effusions: A cost-effective measure in resource-limited settings. Cytojournal 8: 14. 21829416 10.4103/1742-6413.83233 PMC3151400

[37] Tang YC, Liang LB, Yang RX, Cui Y, Qiu HL (2012) Significance of cell block immunohistochemistry in diagnosis of pleural effusions. J Third Mil Med Univ 34: 15.

[38] Bossuyt PM, Reitsma JB, Bruns DE, Gatsonis CA, Glasziou PP, et al (2003) Towards complete and accurate reporting of studies of diagnostic accuracy: the STARD initiative. Standards for Reporting of Diagnostic Accuracy. Clin Chem 49: 1–6. 12507953 10.1373/49.1.1

[39] Whiting P, Rutjes AW, Westwood ME, Mallett S, Deeks JJ, et al (2011) QUADAS-2: a revised tool for the quality assessment of diagnostic accuracy studies. Ann Intern Med 155: 529–536. 22007046 10.7326/0003-4819-155-8-201110180-00009

[40] Deville WL, Buntinx F, Bouter LM, Montori VM, de Vet HC, et al (2002) Conducting systematic reviews of diagnostic studies: didactic guidelines. BMC Med Res Methodol 2: 9. 12097142 10.1186/1471-2288-2-9 PMC117243

[41] Moses LE, Shapiro D, Littenberg B (1993) Combining independent studies of a diagnostic test into a summary ROC curve: data-analytic approaches and some additional considerations. Stat Med 12: 1293–1316. 8210827 10.1002/sim.4780121403

[42] Baker WL, White CM, Cappelleri JC, Kluger J, Coleman CI (2009) Understanding heterogeneity in meta-analysis: the role of metaregression. Int J Clin Pract 63: 1426–1434. 19769699 10.1111/j.1742-1241.2009.02168.x

[43] Deeks JJ, Macaskill P, Irwig L (2005) The performance of tests of publication bias and other sample size effects in systematic reviews of diagnostic test accuracy was assessed. J Clin Epidemiol 58: 882–893. 16085191 10.1016/j.jclinepi.2005.01.016

[44] Zamora J, Abraira V, Muriel A, Khan K, Coomarasamy A (2006) Metadisc: a software for meta-analysis of test accuracy data. BMC Med Res Methodol 6: 31. 16836745 10.1186/1471-2288-6-31 PMC1552081

[45] Whiting P, Harbord R, de Salis I, Egger M, Sterne J (2008) Evidencebased diagnosis. J Health Serv Res Policy 13: 57–63. 10.1258/jhsrp.2008.008025 18806193

[46] McGrath EE, Anderson PB (2011) Diagnosis of pleural effusion: a systematic approach. Am J Crit Care 20: 119–127. 21362716 10.4037/ajcc2011685

[47] Davidson B (2011) The diagnostic and molecular characteristics of malignant mesothelioma and ovarian/peritoneal serous carcinoma. Cytopathology 22: 5–21. 21114695 10.1111/j.1365-2303.2010.00829.x

[48] Niu Y, Huang T, Lian F, Li F (2013) Contrast-enhanced ultrasonography for the diagnosis of small hepatocellular carcinoma: a meta-analysis and meta-regression analysis. Tumour Biol 27. 10.1007/s13277-013-0948-z 23807679

[49] Glas AS, Lijmer JG, Prins MH, Bonsel GJ, Bossuyt PM (2003) The diagnostic odds ratio: a single indicator of test performance. J Clin Epidemiol 56: 1129–1135. 14615004 10.1016/s0895-4356(03)00177-x

[50] Schmidt RL, Factor RE (2013) Understanding sources of bias in diagnostic accuracy studies. Arch Pathol Lab Med 137: 558–565. 23544945 10.5858/arpa.2012-0198-RA

