# Supplementary material for: Diagnostic Accuracy of Ber-EP4 for Metastatic Adenocarcinoma in Serous Effusions: A Meta-Analysis
Source: PLoS One. 2014 Sep 17;9(9):e107741. doi: 10.1371/journal.pone.0107741 (PMC4168227; doi:10.1371/journal.pone.0107741)
Supplement: Table S1 — Summary of the studies included in the meta-analysis. (DOCX) [file pone.0107741.s001.docx]

**Table S1.** Summary of the studies included in the meta-analysis.

| First author-year | Country | Specimen | Cutoff | Clone | Manufacturer | Staining | Antigen retrieval |
| --- | --- | --- | --- | --- | --- | --- | --- |
| Diaz-Arias AA - 1993 | Columbia | Cell blocks | ≥10% cells stained | Ber-EP4 | Dako | SABC | Protease |
| Illingworth AL - 1994 | UK | Smears | Membranous and/or cytoplasmic staining | Ber-EP4 | Dako | Immunofluorescence staining | Heat |
| Shield PW - 1994 | Australia | Cell blocks | Membranous and/or cytoplasmic staining | Ber-EP4 | Dako | SABC | Protease |
| Matter Walstra KW- 1996 | Switzerland | Smears | Membranous and/or cytoplasmic staining | Ber-EP4 | Dako | SABC | Protease |
| Bailey ME - 1996 | America | Cell blocks | Membranous staining | Ber-EP4 | Dako | SABC | Protease |
| Jensen ML - 1996 | Denmark | Cell blocks | Membranous and/or cytoplasmic staining | Ber-EP4 | Dako | SP | Protease |
| Delahaye M - 1997 | Netherlands | Smears | Membranous and/or cytoplasmic staining | Ber-EP4 | Dako | SABC | Protease |
| Nagel H - 1998 | Germany | Smears | Membranous and/or cytoplasmic staining | Ber-EP4 | Dako | APAAP technique | Heat |
| Motherby H - 1999 | Germany | Cell blocks | ≥5% cells stained | Ber-EP4 | Dako | SABC | Protease |
| Bjorn Risberg - 2000 | Norway | Cell blocks | Membranous and/or cytoplasmic staining | Ber-EP4 | Dako | _ | _ |
| Dejmek A - 2000 | Sweden | Smears | Membranous and/or cytoplasmic staining | Ber-EP4 | Dako | APAAP technique | Heat |
| Davidson B - 2001 | Norway | Cell blocks | Membranous and/or cytoplasmic staining | Ber-EP4 | Dako | EnVision™+ Kits | Heat |
| Xiangju Li - 2005 | China | Cell blocks | ≥5% cells stained | Ber-EP4 | Dako | SP | Protease |
| Alaa Afify - 2005 | America | Cell blocks | Membranous staining | Ber-EP4 | Dako | SABC | Heat |
| Politi E - 2005 | Greece | Smears | ≥10% cells stained | Ber-EP4 | Dako | Automated Immunostainer | Heat |
| Wanxin W - 2005 | China | Cell blocks | Membranous and/or cytoplasmic staining | Ber-EP4 | Dako | EnVision™+ Kits | Heat |
| Dejmek A - 2005 | Sweden | Smears | ≥30% cells stained | Ber-EP4 | Dako | APAAP technique | Heat |
| Aerts JG - 2006 | Netherlands | Smears | Membranous and/or cytoplasmic staining | Ber-EP4 | Dako | _ | _ |
| Fang F - 2006 | China | Cell blocks | ≥10% cells stained | Ber-EP4 | NeoMarker | SP | Heat |
| Ueda J - 2006 | Japan | Cell blocks | Membranous and/or cytoplasmic staining | Ber-EP4 | Dako | SABC | Heat |
| Johanna M - 2007 | Netherlands | Cell blocks | Membranous and/or cytoplasmic staining | Ber-EP4 | Dako | PowerVision | Heat |
| Palaoro LA - 2007 | Argentina | Smears | Membranous and/or cytoplasmic staining | Ber-EP4 | Dako | SABC | Heat |
| Saleh HA - 2009 | America | Cell blocks | ≥5% cells stained | Ber-EP4 | Dako | iVIEW DAB kit | Protease |
| Bing Liu - 2010 | China | Smears | ≥10% cells stained | Ber-EP4 | Abcam | PV-6000 kit | Heat |
| McKnight R - 2010 | America | Cell blocks | ≥5% cells stained | Ber-EP4 | Dako | EnVision™+ Kits | Heat |
| Su XY - 2011 | China | Cell blocks | Membranous and/or cytoplasmic staining | Ber-EP4 | Dako | SP | Protease |
| Mingzhi C- 2011 | China | Cell blocks | Membranous and/or cytoplasmic staining | Ber-EP4 | Dako | MaxVision TM kit | Protease |
| Arora R - 2011 | India | Cell blocks | Membranous and/or cytoplasmic staining | Ber-EP4 | Dako | SABC | Heat |
| Yingcheng T - 2012 | China | Cell blocks | Cytoplasmic staining | Ber-EP4 | Dako | SP | Protease |

SABC, Strept Actividin-Biotin Complex; SP, streptavidin-perosidase; APAAP, Alkaline phosphatase-anti-alkaline phosphatase technique.
